# Supplementary material for: DUSP6 is upregulated in metastasis and influences migration and metabolism in pancreatic cancer cells
Source: Sci Rep. 2025 Sep 30;15:33996. doi: 10.1038/s41598-025-12967-8 (PMC12484808; doi:10.1038/s41598-025-12967-8)
Supplement: Supplementary file 2 — Supplementary Material 2 [file 41598_2025_12967_MOESM2_ESM.pdf]

Figure 3E

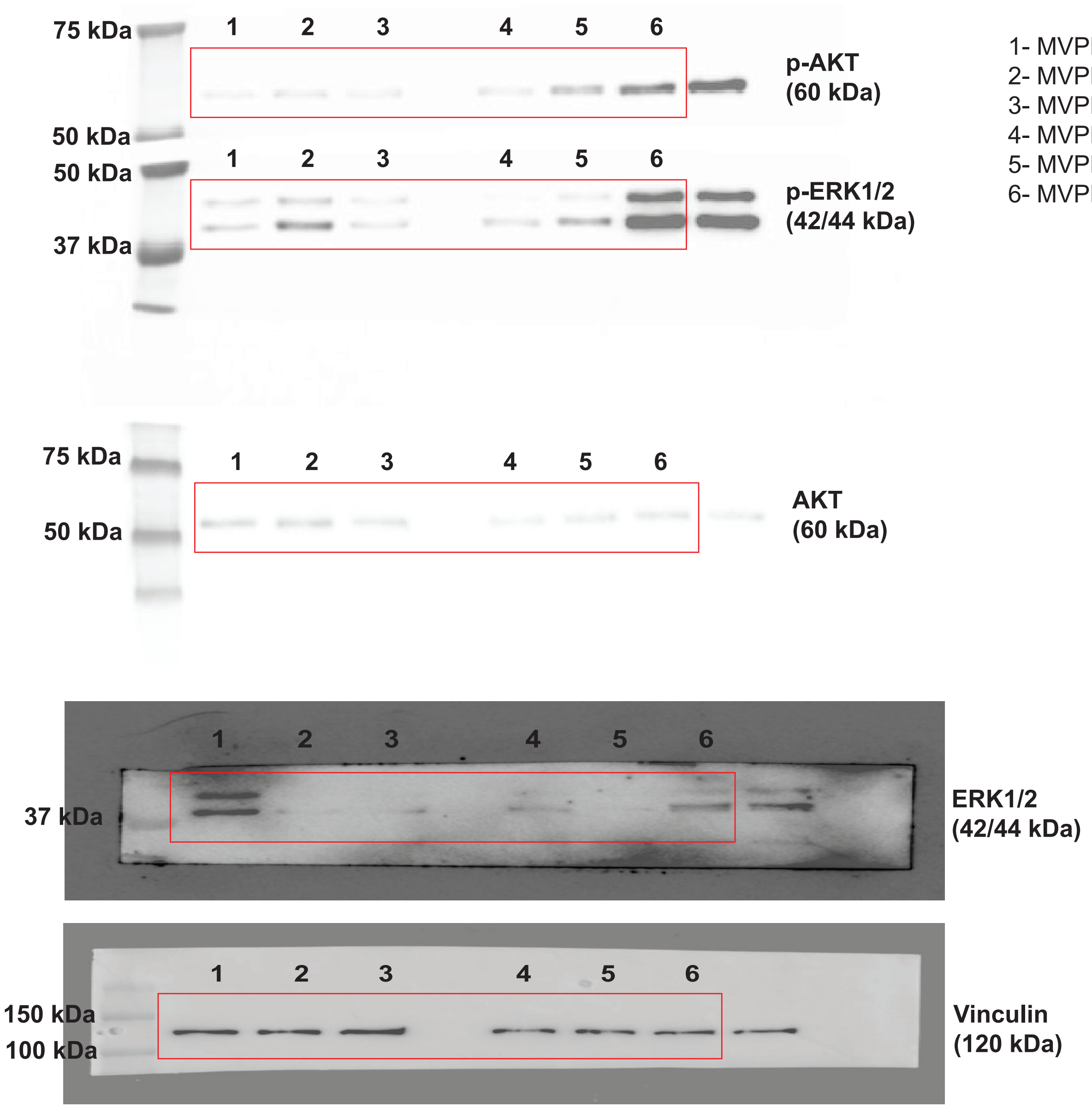

1- MVPMII 48h DMSO
2- MVPMII 24h 5uM BCI
3- MVPMII 48h 5uM BCI
4- MVPMII 90 min DMSO
5- MVPMII 90 min 5uM BCI
6- MVPMII 90 min 50ng/mL TGF-a

Figure S1D

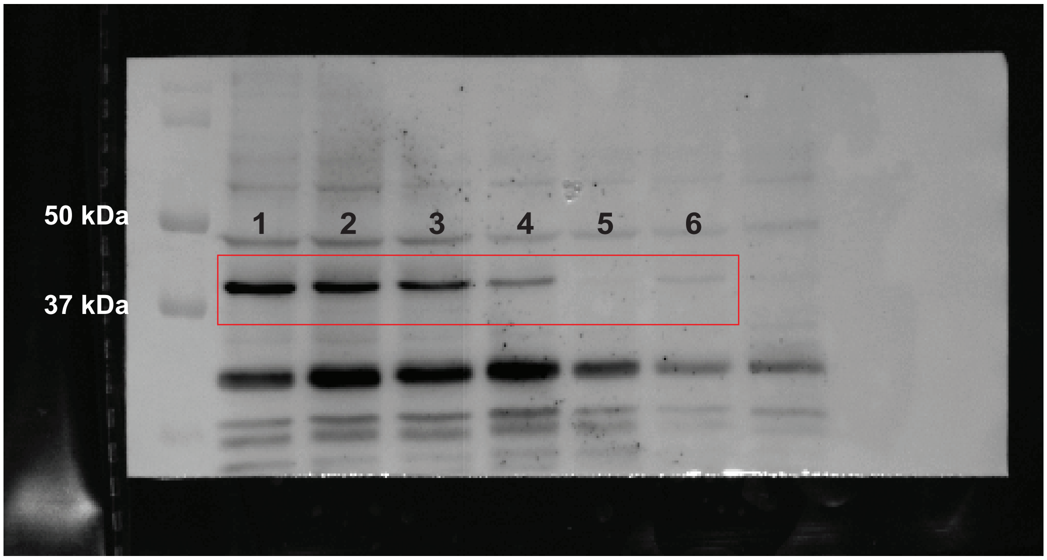

DUSP6 (42 kDa)

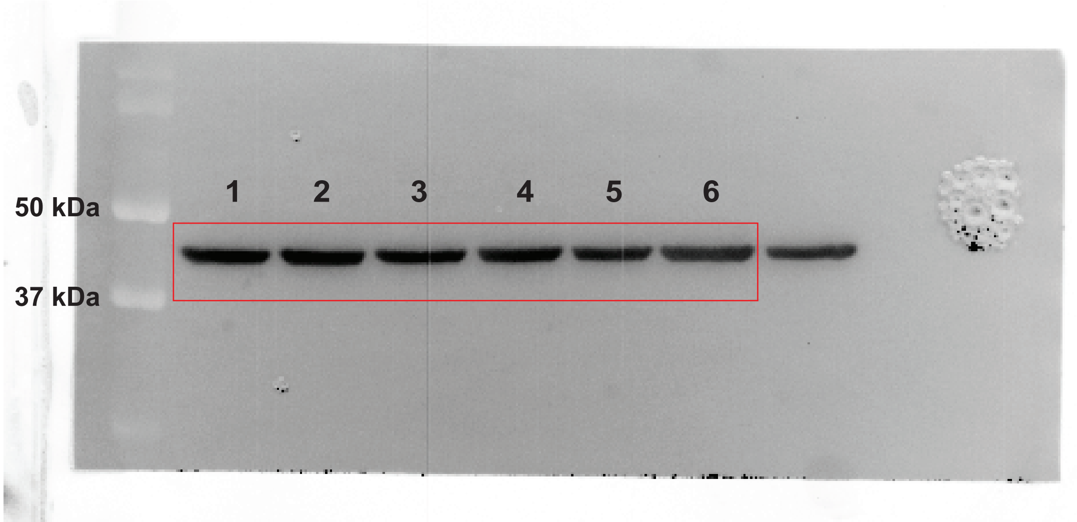

β-actin (42 kDa)

1- DT8082
2- K8484
3- P4313
4- PKT62
5- KPC CAF
6- PKT CAF

Figure S3A

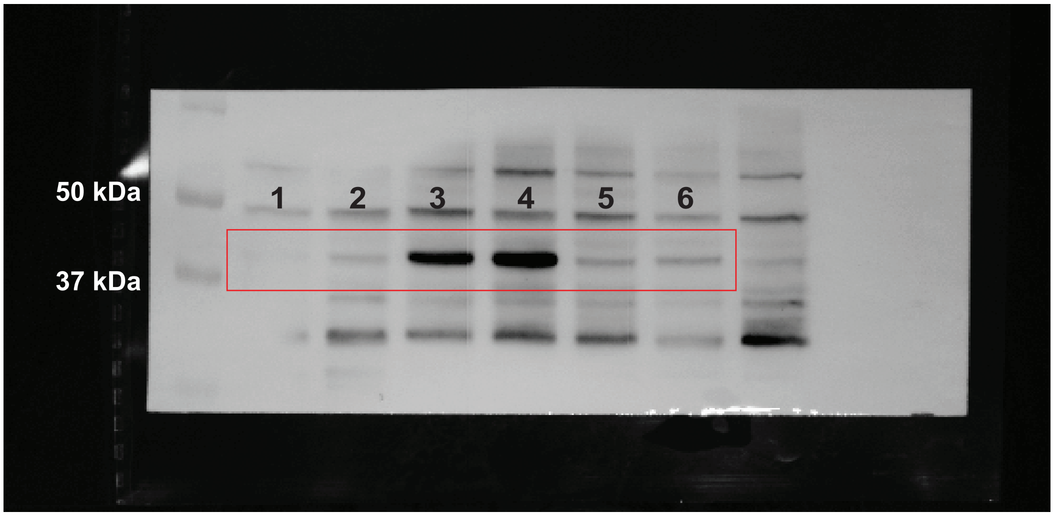

DUSP6 (42 kDa)

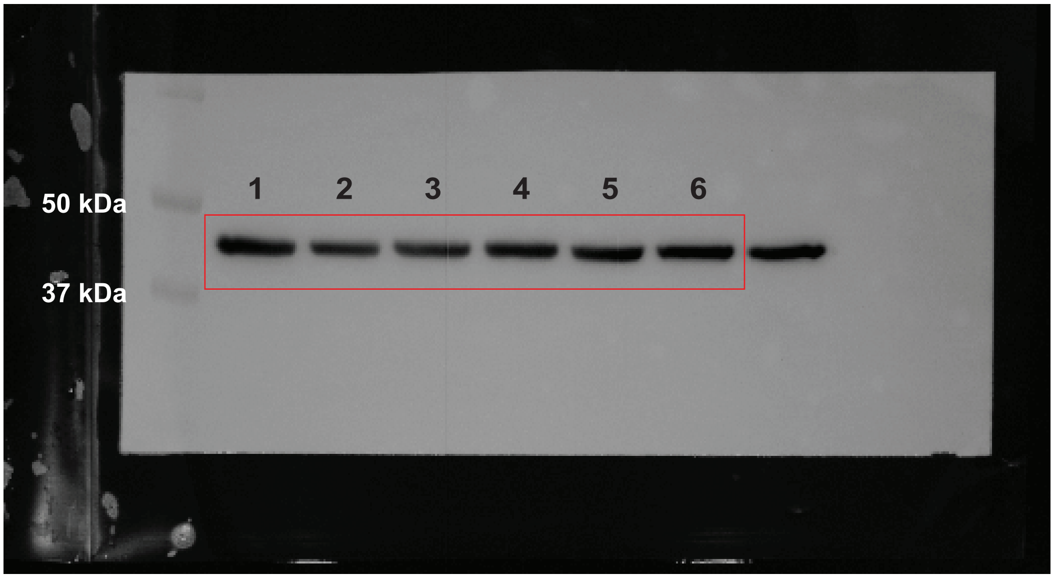

β-actin (42 kDa)

1- MIA PaCa-2
2- PANC-1
3- AsPC-1
4- BxPC-3
5- Capan-2
6- SW1990

Figure S3B

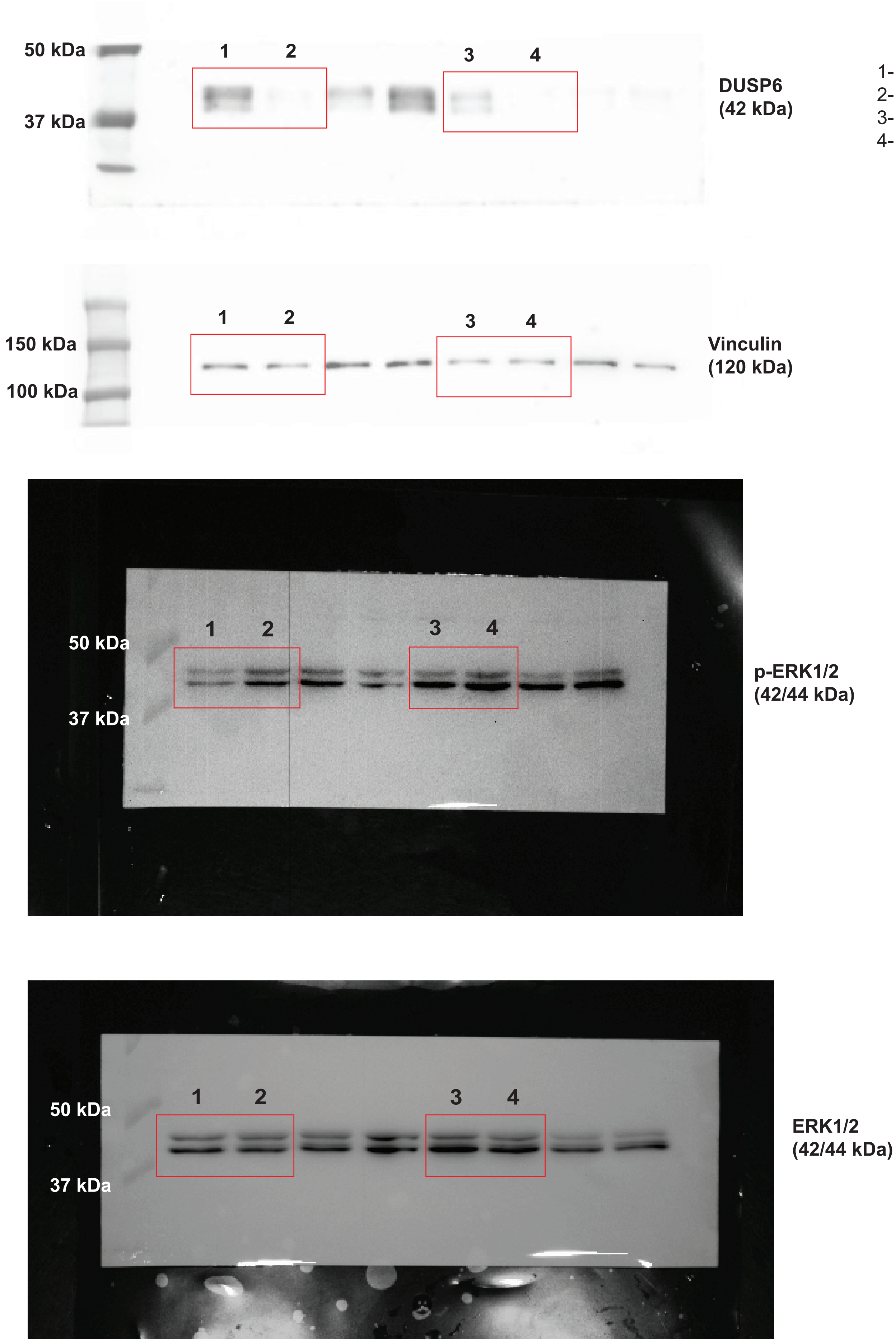

1- AsPC-1 shSCR
2- AsPC-1 shDUSP6 #1
3- BxPC-3 shSCR
4- BxPC-3 shDUSP6 #1

Figure S4E-F

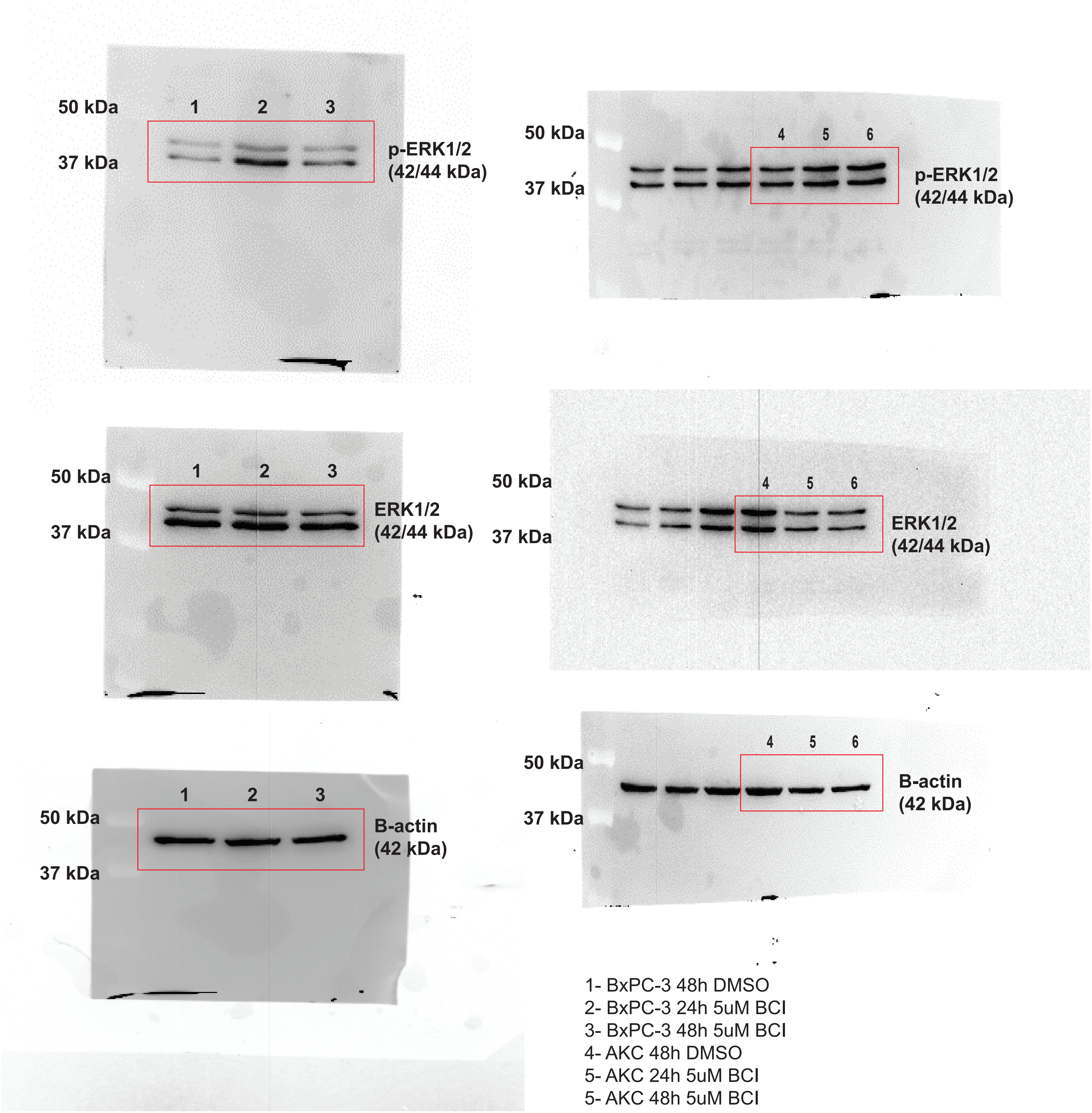

1- BxPC-3 48h DMSO
2- BxPC-3 24h 5uM BCI
3- BxPC-3 48h 5uM BCI
4- AKC 48h DMSO
5- AKC 24h 5uM BCI
5- AKC 48h 5uM BCI

Figure S4G

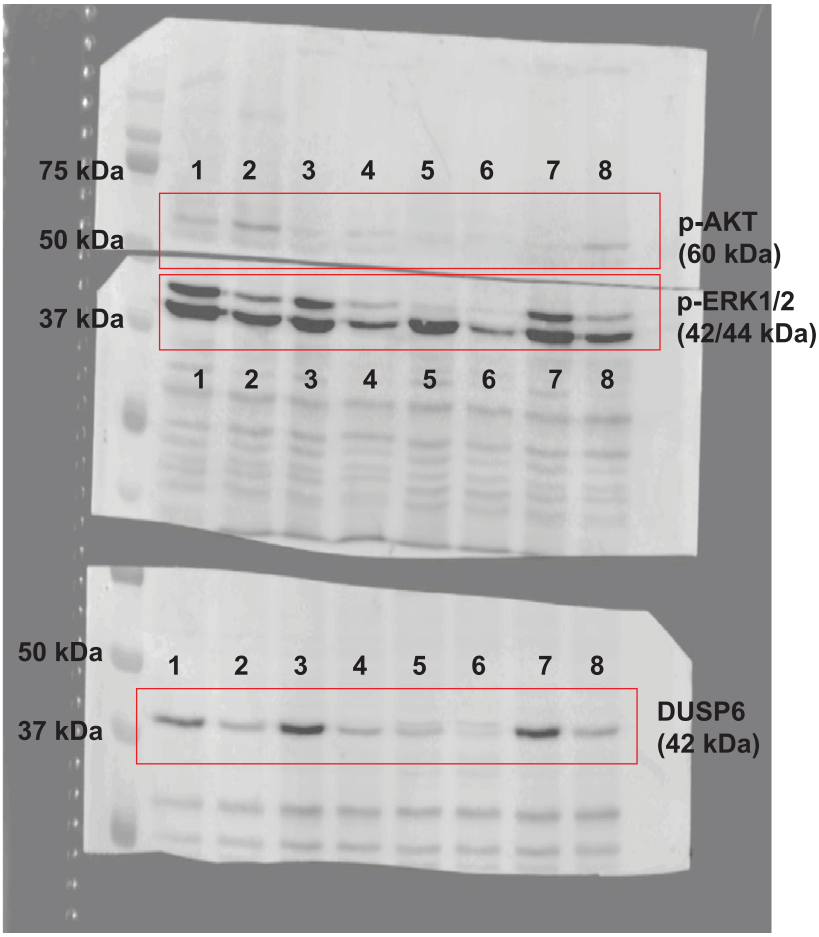

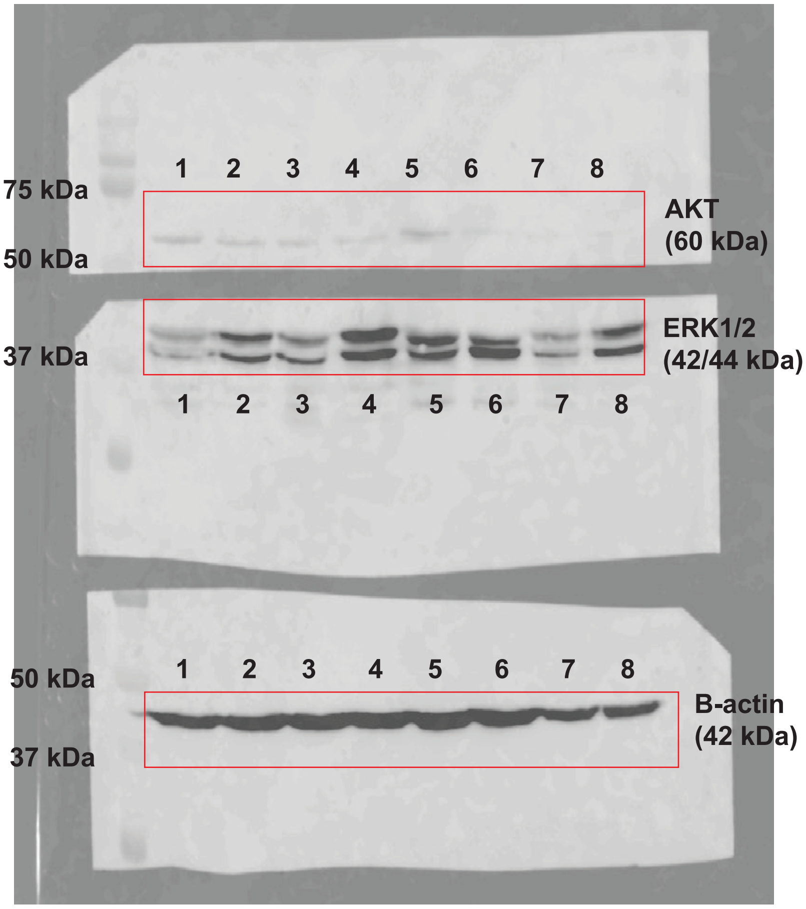

1- K8484 24h DMSO
2- K8484 24h 10nM Trametinib
3- PKT62 24h DMSO
4- PKT62 24h 10nM Trametinib
5- MIA PaCa-2 24h DMSO
6- MIA PaCa-2 24h 20nM Trametinib
7- PANC-1 24h DMSO
8- PANC-1 24h 20nM Trametinib
