# Supplementary material for: DUSP6 is upregulated in metastasis and influences migration and metabolism in pancreatic cancer cells
Source: Sci Rep. 2025 Sep 30;15:33996. doi: 10.1038/s41598-025-12967-8 (PMC12484808; doi:10.1038/s41598-025-12967-8)
Supplement: Supplementary file 3 — Supplementary Material 3 [file 41598_2025_12967_MOESM3_ESM.pdf]

Supplementary figure 1

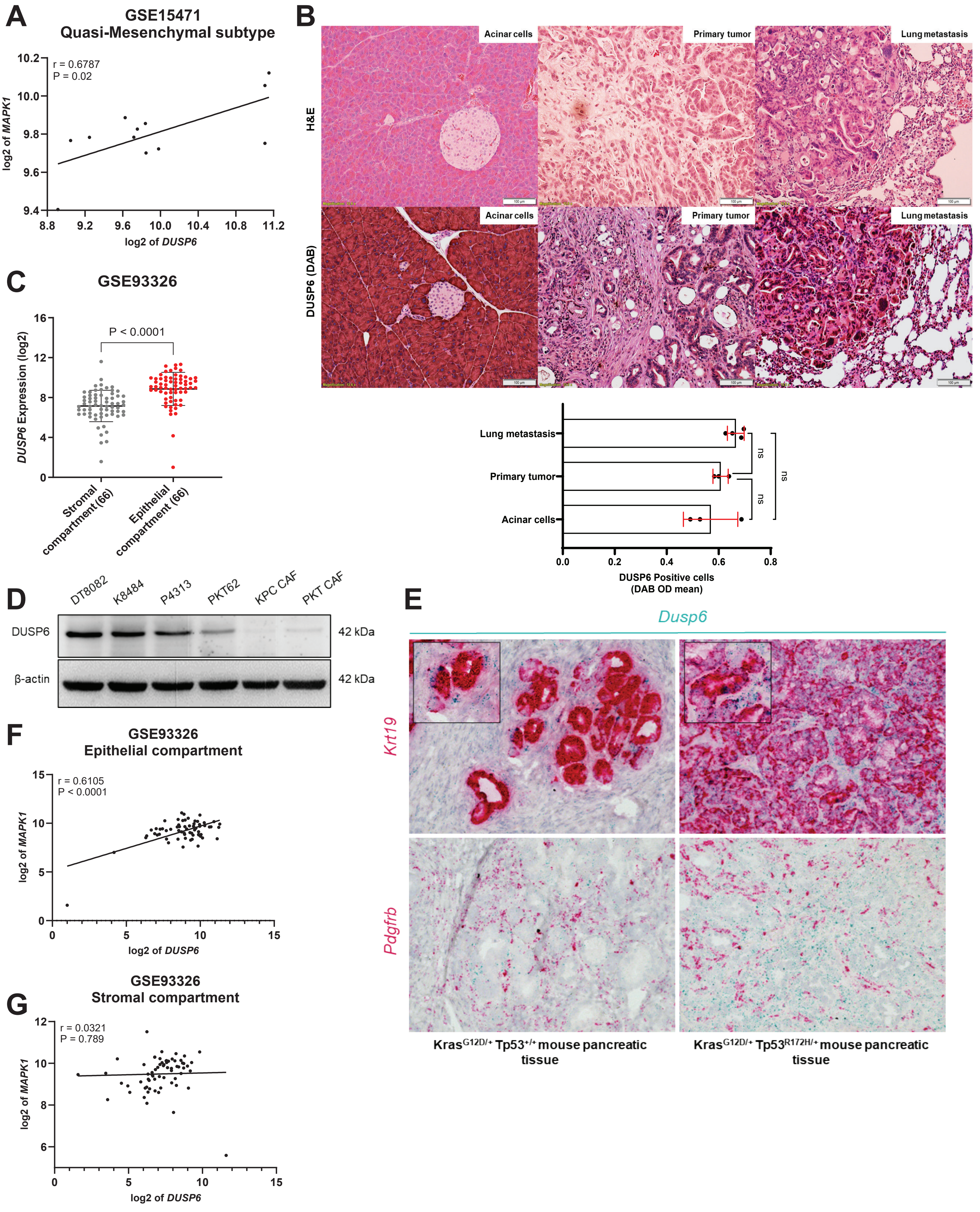

Supplementary figure 2

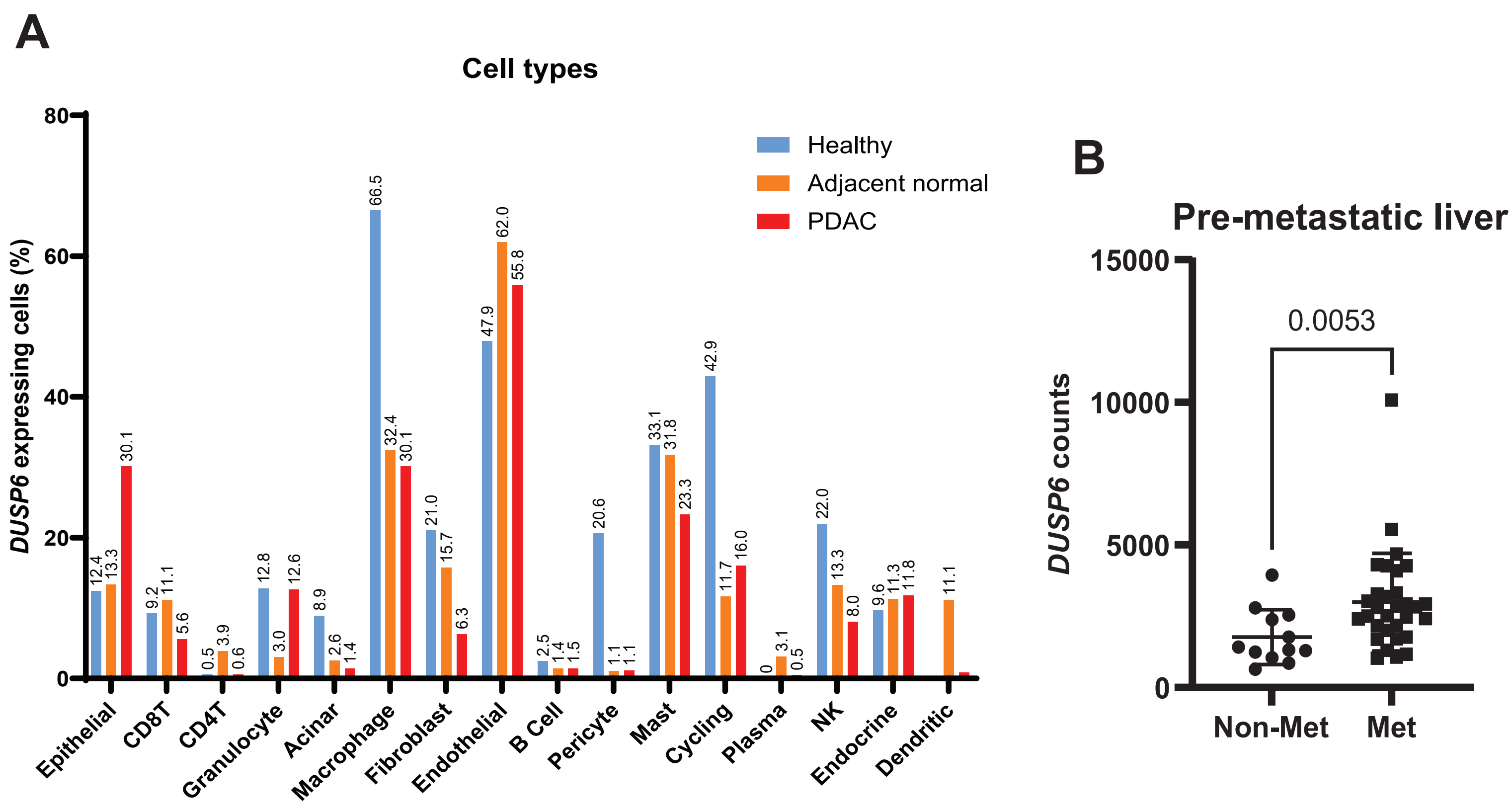

Supplementary figure 3

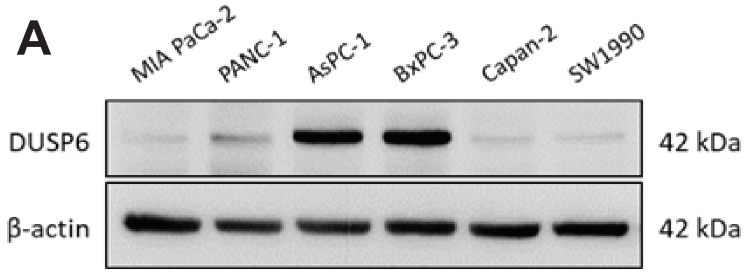

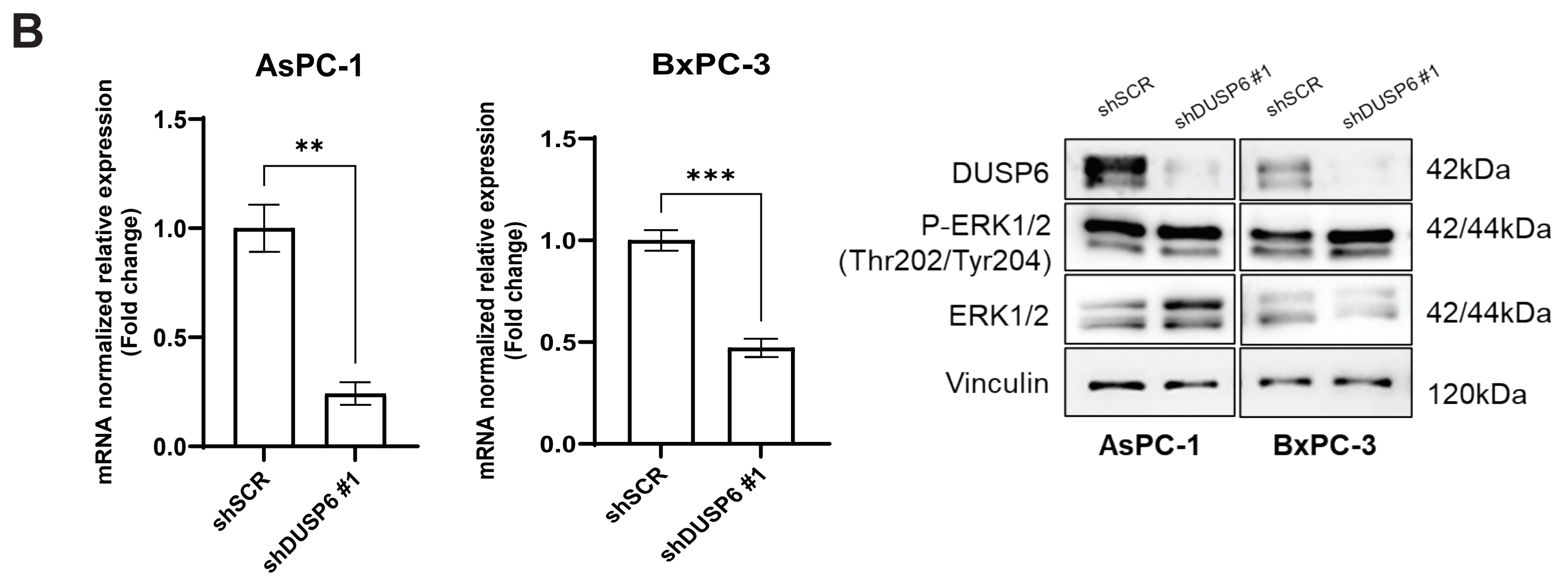

Supplementary figure 4

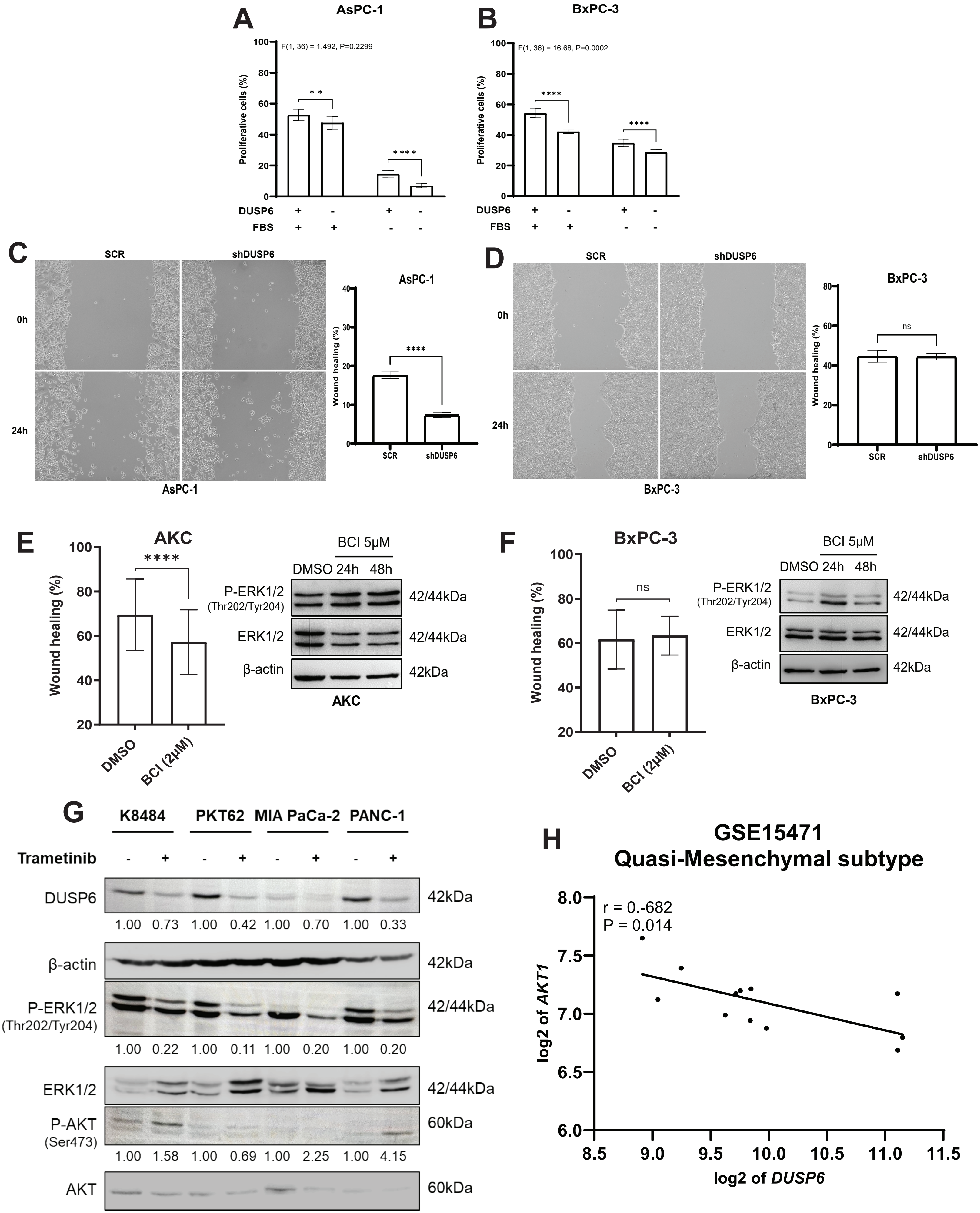

Supplementary figure 5

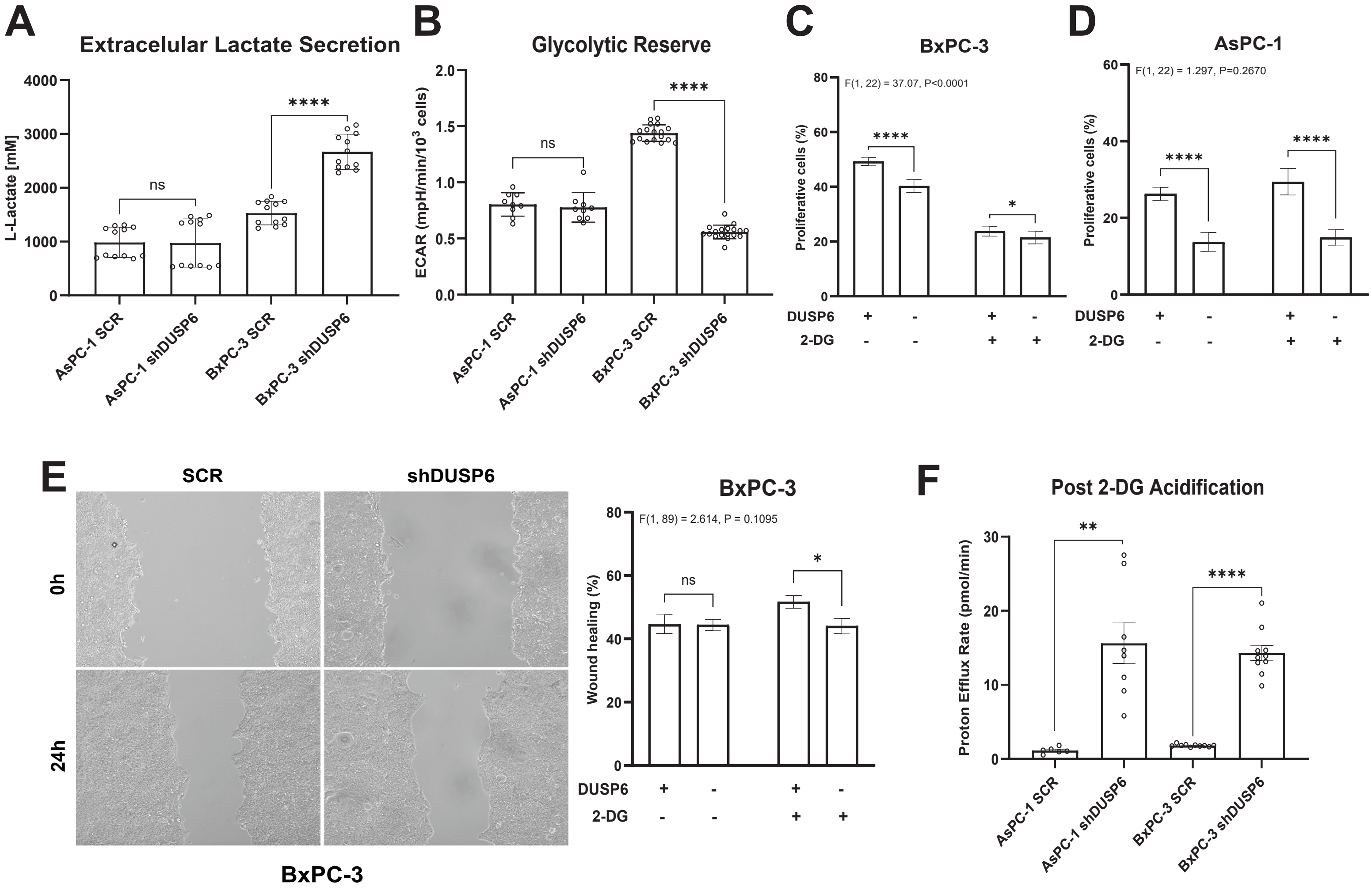

Supplementary figure 6

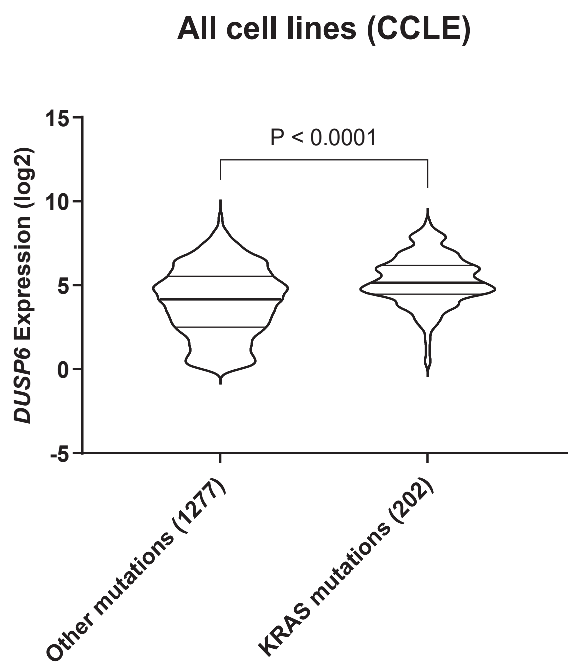

Supplementary Figure 1 – A) Pearson correlation analysis between *DUSP6* expression and *MAPK1* expression using the quasi-mesenchymal subset of GSE15471 [32] dataset; **B)** DUSP6 immunohistochemistry staining (20x magnification) in L-iKras mice. Cre negative littermates were used as controls. Quantification shows DAB OD mean ± SD in DUSP6 positive cells from 5 randomly selected areas in each slide stratified by lesion grade; **C)** *In silico* gene expression analysis utilizing GSE93326 [37] to assess DUSP6 differential expression between the epithelial and the stromal compartment in primary tumor samples. Mann-Whitney unpaired test; **D)** Western blotting analysis assessing DUSP6 protein levels in human tumor cell lines, mouse tumor cell lines and cancer associated fibroblasts (CAF) cell lines; **E)** *Dusp6* expression in tumor sections derived from *LSL-Kras*$^{G12D/+}$, *Ptf1a*$^{Cre/+}$ mouse pancreatic tissue (KC) and *LSL-Kras*$^{G12D/+}$, *Tp53*$^{R172H/+}$, *Ptf1a*$^{Cre/+}$ mouse pancreatic tissue (KPC) using the RNAscope technology. *Dusp6* (green); *Krt19* (red, upper images); *Pdgfrb* (red, lower images); Pearson correlation analysis between *DUSP6* expression and *MAPK1* expression using the epithelial compartment **(F)** and the stromal compartment **(G)** of GSE93326 dataset.

Supplementary Figure 2 – A) *DUSP6* expression among cell types in healthy pancreatic tissue, adjacent normal pancreatic tissue and PDAC samples assessed by single-cell RNA-seq [32]. **B)** *DUSP6* expression was assessed by bulk RNA-seq (GSE245535) in liver biopsy samples from a cohort of patients who underwent pancreatectomy and were followed for 3 years to monitor metastasis development [39]. Mann-Whitney unpaired test.

Supplementary Figure 3 – A) DUSP6 protein levels in a panel of human PDAC cell lines; **B)** Validation of *DUSP6* knockdown in PDAC cell lines. *DUSP6* expression was normalized to *ACTB*. DUSP6 protein levels were normalized to Vinculin levels, while P-ERK1/2 levels were normalized to total ERK levels.

Supplementary Figure 4 – DUSP6 downregulation reduces proliferation and migration in PDAC cell lines *in vitro*. Assessment of proliferation and migratory capacity in AsPC-1 (**A** & **C**) and BxPC-3 (**B** & **D**) with *DUSP6* knockdown; Assessment of migratory capacity AKC **(E)** and BxPC-3 **(F)** upon 24h treatment with BCI (2μM), alongside representative western blots of ERK1/2 activation induced by treatment; **G)** Western blot analysis of ERK1/2 and AKT activation in a panel of human PDAC cell lines upon 24h treatment with Trametinib (10nM K8484 & PKT62, 20nM PANC-1 & MIA PaCa-2). **H)** Pearson correlation analysis between *DUSP6*

expression and *AKT* expression using the quasi-mesenchymal subset of GSE15471 [32] dataset. Graphs show 3 independent experiments with 4-6 replicates each. Results represent mean ± SEM; Brown-Forsythe and Welch ANOVA test. *ns*: not significant; * P < 0.05; ** P < 0.01; *** P < 0.001; **** P < 0.0001.

Supplementary Figure 5 – A) Basal extracellular lactate secretion levels upon *DUSP6* knockdown in complete media; **B)** Glycolytic reserve (difference between glycolytic capacity and basal glycolysis) upon *DUSP6* knockdown. **C & D)** Assessment of proliferation of *DUSP6* knockdown cells upon 2-DG treatment. **E)** Assessment of migratory capacity with 2-DG treatment in BxPC-3 upon *DUSP6* knockdown. **F)** Extracellular acidification rate in PDAC cell lines upon combination of electron transport chain inhibition (Rotenone and Antimycin A) and glycolysis inhibition (2-DG). Seahorse Glycolysis Stress Test was performed in 9-12 replicates in 2 independent experiments. Media contains 1 mM pyruvate, 2 mM glutamine and 10 mM glucose; Results represent mean ± SD of one representative experiment. Welch's unpaired t-test; Proliferation and migration graphs show 3 independent experiments with 4-6 replicates each. Results represent mean ± SEM. Mixed-effects analysis with Šídák's multiple comparisons test. *ns*: not significant; * P < 0.05; ** P < 0.01; *** P < 0.001; **** P < 0.0001.

Supplementary Figure 6 – *DUSP6* expression analysis performed *in silico* on the CCLE dataset. Data shows all the cell lines available on the platform, stratified into cells harboring KRAS mutations and cells that do not.
